# Cross-cultural Comparison of Recovery College Implementation Between Japan and England: Corpus-based Discourse Analysis

**DOI:** 10.1007/s11469-024-01356-3

**Published:** 2024-07-05

**Authors:** Yasuhiro Kotera, Yuki Miyamoto, Sara Vilar-Lluch, Ikuya Aizawa, Owen Reilly, Akihiro Miwa, Michio Murakami, Vicky Stergiopoulos, Hans Kroon, Kirsty Giles, Kennedyrae Garner, Amy Ronaldson, Merly McPhilbin, Tesnime Jebara, Simran Takhi, Julie Repper, Sara Meddings, Jessica Jepps, Adelabu Jonathan Simpson, Vanessa Kellermann, Naoko Arakawa, Claire Henderson, Mike Slade, Shigeyuki Eguchi

**Affiliations:** 1 School of Health Sciences, Institute of Mental Health, University of Nottingham, Nottingham, NG7 2TU UK; 2 Center for Infectious Disease Education and Research, Osaka University, Suita, Osaka 565-0871 Japan; 3 Department of Psychiatric Nursing, Graduate School of Medicine, The University of Tokyo, Bunkyo-Ku, Tokyo, 1130033 Japan; 4 School of English, University of Nottingham, Trent Building, University Park, Nottingham, NG7 2RD UK; 5 RECOLLECT Lived Experience Advisory Panel, London, SE5 8AF UK; 6 Kanagawa Human Rights Advocacy Center for Psychiatric Health, Yokohama, 235-0023 Japan; 7 Department of Psychiatry, University of Toronto, Toronto, Ontario M5T 1R8 Canada; 8 Tranzo, Tilburg School of Social and Behavioral Sciences, Tilburg University, Tilburg, The Netherlands; 9 South London and Maudsley NHS Foundation Trust, Maudsley Hospital, London, SE5 8AZ UK; 10 Nottinghamshire Healthcare NHS Foundation Trust, Duncan Macmillan House, Porchester Road, Mapperley, Nottingham, NG3 6AA UK; 11 Health Service and Population Research Department, Institute of Psychiatry, Psychology and Neuroscience, King’s College London, De Crespigny Park, London, SE5 8AF UK; 12 ImROC, Nottinghamshire Healthcare NHS Foundation Trust, Duncan Macmillan House, Porchester Road, Mapperley, Nottingham, NG3 6AA UK; 13 Division of Pharmacy Practice and Policy, School of Pharmacy, University of Nottingham, Nottingham, NG7 2 UK; 14 Faculty of Nursing and Health Sciences, Health and Community Participation Division, Nord University, Postbox 474, 7801 Namsos, Norway; 15 Tokyo Musashino Hospital, Komone, Itabashi City, Tokyo, 173-0037 Japan

**Keywords:** Recovery College, Japan, England, Corpus-based discourse analysis, Mental health recovery, Cross-culture

## Abstract

Recovery Colleges (RCs) are mental health learning communities, operated in 28 countries across cultures. However, the RC operational model is informed by Western countries sharing similar cultural characteristics such as individualism and short-term orientation. How RC operational model needs to be adapted to non-Western culture remains unknown. We investigated how RCs are introduced to the public in two countries with contrasting cultural characteristics: Japan (collectivism, long-term) and England (individualism, short-term). Corpus-based discourse analysis on 22,827 words from promotional texts (13 RCs in Japan, 61 in England) revealed that both countries emphasised mental illness lived experiences. In Japan, the focus was on the relational and long-term aspects of recovery. In England, the focus was on personal learning and skill acquisition. People attending RCs in Japan may anticipate experiencing collectivistic and long-term elements, which are viewed unfavourably in the operational model. Findings suggest refinements to the operational model to include under-represented cultural characteristics.

**Supplementary Information:**

The online version contains supplementary material available at 10.1007/s11469-024-01356-3.

Mental health recovery has garnered significant attention in mental health communities (Slade et al., 2014). Mental health recovery is commonly defined as “a deeply personal, unique process of changing one’s attitudes, values, feelings, goals, skills and/or roles” and “a way of living a satisfying, hopeful and contributing life even within the limitations caused by illness” (Anthony, 1993). Recovery-oriented approaches have been implemented globally, demonstrating positive impacts such as empowerment, hope, autonomy and reduced stigma among service users (Dell et al., 2021; Ellison et al., 2018). These approaches also fostered positive attitudes and a better understanding of service users among mental health staff (McPherson et al., 2021). Mental health recovery principles have been integrated into numerous national policies (Department of Health & Ageing, 2009; Department of Health Social Services & Public Safety of Northern Ireland, 2018; Department of Mental Health-Thailand, 2019; HM Government, 2011; Mental Health Commission of Canada, 2012; New Freedom Commission on Mental Health, 2003; Slade et al., 2012).

Recovery Colleges (RCs) are a relatively new recovery-oriented approach providing mental health service users, carers and staff with social support and opportunities to develop skills. The concept of RCs was inspired by education centres and peer-run services (services that were run by people with lived experience) for mental health recovery, operated in the United States during the 1990s (Slade et al., 2014). The first RC was established in England in 2009. Since then, RCs have spread to 28 countries across economic levels and cultural characteristics, and the service settings have been diversified, e.g. primary and secondary mental healthcare, non-governmental organisations and education providers (Hayes et al., 2023).

RCs are regarded as a mental health innovation (Whitley et al., 2019). Distinctive features of RCs are two key approaches of co-production and adult education. Co-production is commonly defined as the involvement of lived experience and professional expertise in planning, designing, delivery and quality assurance of the programmes (Toney et al., 2018b). Adult education refers to self-directed learning that is characterised as strengths-based, person-centred, inclusive and community-focused (Thériault et al., 2020; Toney et al., 2018a). Co-production and adult learning enable personal recovery, which means living a purposeful and autonomous life despite the presence of mental health symptoms (Slade et al., 2014). Co-production is built upon two conceptual shifts related to personal recovery: (a) care focuses on the person, instead of the symptoms, and (b) empowerment (i.e. having control over one’s own life) and quality of life are as important as symptom reduction (Lin et al., 2022a, 2022b). Therefore, in the RC model, recovery is realised through social inclusion of the students such as gaining or increasing social and/or economic roles. Anybody, including people with mental health symptoms, as well as formal and informal carers, can register as students in a RC (Lin et al., 2022a, 2022b; Perkins et al., 2012). RCs aim to help students manage their own wellbeing. e.g. coping with anxiety, and anger management (Kelly et al., 2017). Courses are intended to support students to understand recovery, rebuild their life (e.g. goal setting), develop life skills (e.g. money management) and get more involved in a RC (e.g. becoming a peer trainer) (Meddings et al., 2015).

The evidence about RC effectiveness and cost-effectiveness has been promising. Reviews and qualitative evidence synthesis about the impacts of RCs identified benefits on students and staff and cost-effectiveness (Thériault et al., 2020; Toney et al., 2018a; Whish et al., 2022). Benefits for students include increased confidence, hope, quality of life, empowerment and reduced stigma. Benefits for staff include enhanced motivation, skills and knowledge, which contribute to positive attitudinal changes to co-production and service users. For cost-effectiveness, RC attendance was associated with less in-patient days, unintended hospital admissions and community contacts over 18 months (Bourne et al., 2018). Reduced service use derived from RC attendance accounted for net savings of Australian $269 per student per year (Cronin et al., 2021).

Evidence for cross-cultural differences remains under-developed in RCs. This is concerning because RCs are operated in many different cultural contexts around the world. Most RC research has been conducted in Western, educated, industrialised, rich and democratic (WEIRD) countries, lacking evidence from other countries (Hayes et al., 2022; Whitley et al., 2019). In RC research, six reviews have been published to date, which included 186 studies in total (Bester et al., 2022; Crowther et al., 2019; Lin et al., 2022a; Thériault et al., 2020; Toney et al., 2018a; Toney et al., 2018b) (Supplementary Material 1). However, no empirical study has specifically focused on RCs in non-WEIRD countries. Cross-cultural understanding is essential to inform cultural adjustment of the RC operational model to non-WEIRD contexts. Meta-analyses reported notable effect differences between culturally adapted treatment and non-adapted treatment, including a five-time greater likelihood of symptom remission with culturally adapted treatment (Arundell et al., 2021; Hall et al., 2016; Rathod et al., 2018). Differences in operation between RCs in non-WEIRD and WEIRD countries remain unknown.

This study compared how RCs in Japan and England are introduced to the public, by evaluating promotional texts. Recent global studies on RCs have identified that the current RC operational model, informed by the RC components (Supplementary Material 2), is more aligned with cultural characteristics of WEIRD countries as defined in Hofstede’s cultural dimensions theory (Hofstede & Minkov, 2013). The theory presents a framework for understanding cultural differences across countries, and how these differences impact people’s behaviours based on six characteristics. Of the six cultural characteristics, the ones associated with the current RC operational model are individualism, uncertainty acceptance, indulgence and short-term orientation (no significant associations with power distance and success-drivenness) (Hayes et al., 2023; Kotera et al., 2024a, 2024b). Individualism refers to a degree to which a society expects individuals to take care of only themselves and their immediate family. Uncertainty acceptance means a degree to which individuals feel comfortable with unknown situations. Indulgence is acceptance of relatively free gratification of basic human needs, rather than controlling them. Short-term orientation values immediate results rather than future rewards (Hofstede & Minkov, 2013). The current RC operational model considers RCs oriented towards these four cultural characteristics to be more aligned with the model compared to other RCs. Compared with WEIRD cultures, Japanese culture is characterised as the opposite of these four characteristics: collectivism, uncertainty avoidance, self-restraint and long-term orientation. Culturally adapted promotional texts are crucial to healthcare implementation success (Elrod & Fortenberry, 2020; Lugovoy & Lugovaya, 2019). Healthcare providers create promotional texts that reflect the culture of the public to maximise engagement (Larkey & Hecht, 2010). However, no studies have evaluated RC promotional texts to date.

Our research questions (RQs) were as follows:

1. How are RCs introduced in promotional texts to the public in Japan and England?
2. How do linguistic differences in the promotional texts relate to cultural differences between the two countries?

## Material and methods

### Design

This was a text-based qualitative and quantitative study to analyse RC promotional texts that were publicly available in Japan and England. The study aimed to identify similarities and differences between RCs in Japan and England. Ethics committee approval was not required for this study.

### Materials

The online software Sketch Engine (Kilgarriff et al., 2014) was used for the linguistic analysis. Sketch Engine helps understand how words and phrases are used in the real-word language by providing detailed information about textual context, frequencies and language patterns (Kilgarriff et al., 2014) (see Supplementary Material 3 for a list of methodological terms).

### Data collection

Promotional texts were collected from RC websites. Texts in 61 RCs in England (Supplementary Material 4) were collected by co-authors YK and SVL (11 October to 19 November 2023). Texts in all 13 RCs in Japan were collected by co-authors YK and YM (26 September to 11 October 2023; Supplementary Material 5). Japanese texts were first translated into English using the DeepL Translator (DeepL, 2017), then were checked by YK, a professional translator versed in RC terminology. Japanese text data comprised 813 words after translation into English. Text data from RCs in England comprised 22,014 words (Supplementary Material 4). A data sub-set of RCs in England with a similar word count to that of RCs in Japan was built to avoid skewed results in statistical comparisons (Table 1). The data sub-set of RCs in England and the rest of the English data were linguistically similar: corpus similarity 0.60 of 1.00 (Dunn, 2020; Kilgarriff, 2001).

**Table 1:** Japan dataset and England data sub-set with word counts

Japan	Words	England	Words
RC Mitaka	113	Digital RC	288
RC Ohta	113	Severn and Wye RC	243
RC Kochi	94	Oxfordshire RC	231
RC Kobe	88	Leicestershire RC	89
RC Tanto	88		
RC Okayama	75		
RC Fukuoka	62		
RC Nagoya	50		
RC Saga	45		
RC Annaka	37		
RC Mimasaka	21		
RC Neyagawa	0		
Total	813		851

### Data analysis

We performed a corpus-based discourse analysis (Flowerdew, 2023). Corpus analysis (Hunston, 2002) involves the software-assisted examination of large collections of digitised texts (“corpus”), with typical sizes ranging from millions to billions of words (e.g. British National corpus, news on the Web Corpus). Our discourse analysis was informed by the critical discourse analysis tradition (Fairclough, 2010, 2014). An adapted version of the framework (Mullet, 2018) used in this study is provided in Supplementary Material 6. Textual analysis was performed using common corpus tools to allow for quantitative comparisons: (a) wordlist, (b) word sketch and (c) keywords analyses. Interpretation of textual analysis as reflecting sociocultural practices was informed by (a) Hofstede’s cultural dimensions theory (Hofstede & Minkov, 2013) and (b) an RC study about the impact of culture on the operational model, reflecting characteristics of context of production (RCs) and values of producers and audience (RC managerial staff and students) (Kotera et al., 2024a, 2024b).

1. Wordlist compiles lists of words from texts and identifies the raw frequency (RaF: number of occurrences) of each word in a dataset. To allow comparisons between the datasets, relative frequency (ReF) was calculated. ReF is the ratio between the RaF of a word and the total number of words in the dataset, expressed per 1000 words (1000 was chosen as being close to the data amounts: 813 words in Japan, 851 in England). We examined the top 10 verbs (processes), nouns (entities) and adjectives (descriptive terms) per dataset.
2. Word sketch automatically extracts “collocates”—words co-occurring with a focus word with a frequency higher than chance—and shows patterns of use of the focus word and the collocates. We set “recovery” as the focus word and examined how “recovery” was introduced in each dataset.
3. Keyword analysis involves the automatic extraction of statistically significant words in a dataset, as compared to a reference dataset (Culpeper & Demmen, 2015). Keywords reflect the uniqueness of a dataset (“keyness”). The England data sub-set was used as reference for extracting keywords of the Japan dataset and vice versa. This process identified keywords of the Japanese RCs, compared to the English RCs, and vice versa. Analysis of both single and multi-term keywords enabled us to distil key themes from each dataset, with themes identified based on total keyness scores (TKSs) exceeding 10,000. We examined the top 50 keywords and focused on lexical words (e.g. nouns, verbs), which were content-informative compared with grammatical words such as prepositions.

The contexts of use of keywords and high frequency terms were examined with the concordance tool, allowing to examining word and phrase usage in context (Wynne, 2008), to identify themes inductively.

## Results

### Overview

Wordlist analysis showed similarities between Japan and England (full) datasets in relation to entities being talked about (nouns), processes attributed to them (verbs) and descriptive words (adjectives) (Table 2). High-frequency terms, assisted by concordance checks, identified seven themes: personal learning; place for wellbeing; recovery; mental illness experience; reference to service user; education; and community.

**Table 2:** Wordlists with raw and relative frequencies for top 10 verbs, nouns and adjectives in Japan and England RCs (full dataset)

Theme	Word type	Japan (813 words)			England (22,014 words)		
			Raw frequency	Relative frequency		Raw frequency	Relative frequency
Personal learning	Verb (processes)	**Learn**	19	23.37	**Learn**	115	5.22
	Total		19	23.37		115	5.22
Place for wellbeing	Nouns (entities)	Place	15	18.45	**College**	220	9.99
		**College**	14	17.22			
	Total		29	35.67		220	9.99
Recovery	Verb (processes)	Improve	2	2.46			
	Nouns (entities)	**Recovery**	37	45.51	**Recovery**	414	18.81
	Adjectives (descriptive words)	Rich	2	2.46	Personal^a^	33	1.49
	Total		41	50.43		447	20.30
Mental illness experience	Nouns (entities)	**Experience**	9	11.07	**Experience**	167	7.59
		Difficulty	8	9.84			
		Illness	6	7.38			
	Adjectives (descriptive words)	**Mental**	17	20.91	**Mental**	283	12.86
		**Personal** 2 2.46			Physical	37	1.68
					**Personal**^**a**^	14	0.64
	Total		42	51.66		501	22.76
Reference to service user	nouns (entities)	**People**	10	12.30	**People**	230	10.45
					Student	107	4.86
	Total		10	12.30		337	15.31
Education	verb (processes)				Work	86	3.91
					Offer	84	3.82
					Support	80	3.63
					Provide	70	3.18
	Nouns (entities)	knowledge	4	4.92	Course	315	14.31
					Service	90	4.09
	Adjectives (descriptive words)				Educational	48	2.18
					Free	42	1.91
					Professional	32	1.45
	Total		4	4.92		847	38.48
Community	Verb (processes)	Gather	2	2.46			
	Total		2	2.46		0	0.00

Similarities (i.e. words common between RCs in England and Japan) are presented in bold

^a^The concordance tool revealed that “personal” in the England dataset describes the “recovery” and “mental illness experience” aspects of RC. Words that were not associated with the coding scheme are not included

Frequency analysis was complemented with a keywords analysis (single and multi-terms) to examine those themes that were uniquely emphasised in the Japanese RC dataset, and in the English RC data sub-set (Table 3). “Mental illness experience” was highlighted in both countries. In Japan, “Place for wellbeing”, “recovery” and “community” were uniquely highlighted. In England, “personal learning” and “education” were uniquely highlighted.

**Table 3:** Single and multi-term keywords for RCs in Japan and England

	Japan (813 words)				England (851 words)			
Theme	Single (raw frequency/keyness score)	Total keyness score	Multi-term (raw frequency/keyness score)	Total keyness score	Single (raw frequency/keyness score)	Total keyness score	Multi-term (raw frequency/keyness score)	Total keyness score
Personal learning (E)		0.00	Expert in the experience (1/1080.91), expertise of mental health (1/1080.91), life learn (1/1080.91), specific knowledge (1/1080.91)	4323.64	Develop (3/3100.17), skill (3/3100.17), self-management (2/2067.12), ourselves (2/2067.12), become (2/2067.12), style (1/1034.06)	**13,435.76**	Developing skill (1/1034.06), active role (1/1034.06), tried-and-test (1/1034.06), people learn (1/1034.06), developing knowledge (1/1034.06), own resourcefulness (1/1034.06)	6204.36
Place for wellbeing (J)	Where (8/8640.31), bring (4/4320.65), welfare (3/3240.74), provider (2/2160.83), option (1/1080.91), peace (1/1080.91)	**20,524.35**	Health welfare (2/2160.83), welfare service (2/2160.83), health welfare service (2/2160.83), mental health welfare (2/2160.83), mental health welfare service (2/2160.83), care provider (1/1080.91), medical care (1/1080.91), medical care provider (1/1080.91), welfare professional (1/1080.91), welfare service provider (1/1080.91), mental health welfare service provider (1/1080.91)	**17,289.61**	Site (2/2067.12), welcome (1/1034.06), hospital (1/1034.06)	4135.24	Mental health service (3/3100.17), wellbeing recovery college (1/1034.06)	4134.23
Recovery (J)	Wisdom (4/4320.65), process (4/4320.65), rich (2/2160.83), improve (2/2160.83), regain (2/2160.8), hope (2/2160.83), functioning (1/1080.91), happen (1/1080.91)	**20,524.35**	Living life (2/2160.83), improvement in mental symptoms (1/1080.91), cure of an illness (1/1080.91), different recovery (1/1080.91), unique way (1/1080.91), growing movement (1/1080.91), process of recovery (1/1080.91), finding new meaning (1/1080.91), small experience of recovery (1/1080.91)	**10,808.11**		0.00	Wellbeing recovery (1/1034.06), development of new meaning (1/1034.06), contributing life (1/1034.06)	3102.18
Mental illness experience (JE)	Difficulty (8/8640.31), disability (3/3240.74), living (2/2160.83), symptom (2/2160.83), prognosis (1/1080.91), disorder (1/1080.91), psychiatric (1/1080.91), addition (1/1080.91)	**20,526.35**	Health difficulty (3/3240.74), mental health difficulty (3/3240.74), difficulty in living (2/2160.83), various difficulties (2/2160.83), experienced mental health (1/1080.91), experienced mental health hardship (1/1080.91), experienced mental health difficulty (1/1080.91), variety of experiences (1/1080.91), people with difficulties in living (1/1080.91), mental disorder (1/1080.91), mental hardship (1/1080.91), experience of mental health difficulties (1/1080.91), mental symptom (1/1080.91)	**20,531.33**	Story (2/2067.12), diagnosis (2/2067.12), issue (2/2067.12), problem (2/2067.12), limitation (1/1034.06)	9302.54	Mental health problem (2/2067.12), mental health related issue (2/2067.12), health problem (2/2067.12), experience of mental health problems (2/2067.12), health related issue (2/2067.12), catastrophic effect (1/1034.06), experiencing mental health (1/1034.06), experiencing mental health challenge (1/1034.06), catastrophic effect of mental illness (1/1034.06), health condition (1/1034.06), particular diagnosis (1/1034.06), person with mental illness (1/1034.06), effect of mental illness (1/1034.06), personal experience of mental illness (1/1034.06)	**19,642.14**
Reference to service user	Walk (2/2160.83), participant (1/1080.91)	3241.74	Walk of life (2/2160.83)	2160.83		0.00	Service user (4/4133.23), user of the mental health (1/1034.06)	5167.29
Education (E)	Facilitate (1/1080.91), education (1/1080.91)	2161.82	Educational option (1/1080.91), called professional (1/1080.91), mental health professional (1/1080.91)	3242.73	Work (5/5166.29), approach (4/4133.23), provide (4/4133.23), trainer (3/3100.17), aim (3/3100.17), training (2/2067.12), study (2/2067.12), topic (2/2067.12), understanding (2/2067.12) free (2/2067.12), both (2/2067.12), teach (2/2067.12), range (2/2067.12), development (1/1034.06)	**37,204.11**	Lived experience (4/4133.23), lived experience of mental health (2/2067.12), people with lived experience (2/2067.12), called peer (1/1034.06), offering online course (1/1034.06), classroom course (1/1034.06), education course (1/1034.06), thorough perspective (1/1034.06), group discussion (1/1034.06), educational workshop (1/1034.06), health recovery training (1/1034.06), emphasis on sharing stories (1/1034.06)	**17,574.01**
Community (J)	Community (4/4320.65), supporter (3/3240.74), gather (2/2160.83), movement (2/2160.83), social (2/2160.83), respect (1/1080.91)	**15,124.79**	Learning community (1/1080.91), life in the community (1/1080.91), community member (1/1080.91)	3242.73	Carer (4/4133.23), relate (3/3100.17), co-produce (2/2067.12)	9300.52	Carer of service (1/1034.06)	1034.06

Total keyness score > 10,000 as a key theme in bold. Keywords are classified by theme and ranked by keyness score, which is how a word is statistically distinctive within one dataset compared to the reference dataset (see Kilgarriff, 2001; Kilgarriff et al., 2014). Keyness score = *f*_focus_ + *N* / *f*_ref_ + *N*. *f*_focus_ is the normalised (per million) frequency of the word in the focus corpus, *f*_ref_ is the normalised frequency of the same word in the reference corpus, and *N* the smoothing parameter (by default, *N* = 1). Keywords not coded by theme and references to locations of specific RCs have not been included (e.g. “Oxfordshire”)

(*J*) key theme in the Japan dataset, (*E*) key theme in the England sub dataset

### Personal learning

“Personal learning” characterised RCs in England (Table 3). Data in this theme emphasised service users developing knowledge, skills and self-management. Since “learn” (verb) was identified as recurrent in both datasets (Table 2), concordances for learn (including both the lemma, i.e. the standard form, learn, and derived forms, e.g. “learning”) were examined to identify further differences. Table 4 includes 10 random concordances. RCs in Japan converge individual learning with community experience (examples 1–5 (E1-5)). While references to communal learning were also identified in RCs in England, these references emphasised service users’ individual learning (E6–8) and the educational focus of the RCs (E9–10).

**Table 4:** Concordances for “learn”

Country	Examples—learn (lemma)	
Japan	1	RC is a learning community where anyone can participate as a student, regardless of position
	2	…people with various difficulties and differences gather, bring their wisdom and experiences, and learn from each other to improve their own recovery and mental health
	3	… those who feel difficulty in living, and those who support them can learn from each other in order to face themselves with sincere joy and live a rich life in the community
	4	Recovery College is expanding as a place where people with illnesses and their supporters can learn from each other the knowledge they need to live well in the community
	5	… bring their expertise, knowledge, and wisdom to create a place where they can learn from each other
England (full dataset)	6	… educational courses to help students better understand mental health issues, learn self-management techniques and gain skills to give them better hopes for the future
	**7**	Learn about yourself and your recovery through a unique range of courses and short sessions …
	8	… taking responsibility for personal wellbeing and learning to live alongside any continued symptoms or impairments …
	9	… to make sure that lots of voices contribute to each course, workshop and learning opportunity that we offer
	10	This way, every one of our courses, workshops and learning opportunities are fully designed with Recovery in mind

### Place for wellbeing

RCs as a “place for wellbeing” were particularly emphasised in RCs in Japan (Table 3). Concordances for “college” were recurrent in both datasets (Table 2) and were further examined focusing on characterisations of RCs. Table 5 includes 10 random concordances for “college is”. Both datasets describe college as a space for learning about recovery (E11–15, 17, 19), reflecting education as a path to recovery. RCs in Japan regarded colleges as a space for community (E12–15). While RCs in England also identified colleges as a communal space (E20), their emphasis was placed on RCs being a space for personal development (E16–19).

**Table 5:** Concordances for “college”

Country	Examples—“college is”	
Japan	11	Recovery College is a place to learn mental health recovery on one's own initiative to enrich one’s life…
	12	Recovery College is a place where people with various difficulties and differences gather, bring their wisdom and experiences…
	13	Recovery College is a place to learn together about recovery
	14	Recovery College is expanding as a place where people with illnesses and their supporters can learn from each other…
	15	Recovery College is neither a place of treatment nor a place of support, but rather a place of “proactive learning about recovery”…
England (full dataset)	16	…Recovery College is a safe space where you can be yourself, free from judgement
	17	The Recovery College is all about providing a relaxed and informal educational approach to wellbeing and recovery for people…
	18	The College** is** based on the principles of hope, control and personal responsibility
	19	The college** is** not somewhere to obtain qualifications but to learn self-management and personal development
	20	The College is open to people with mental health challenges, their relatives, friends and carers and to staff…

### Recovery

**Fig. 1:**
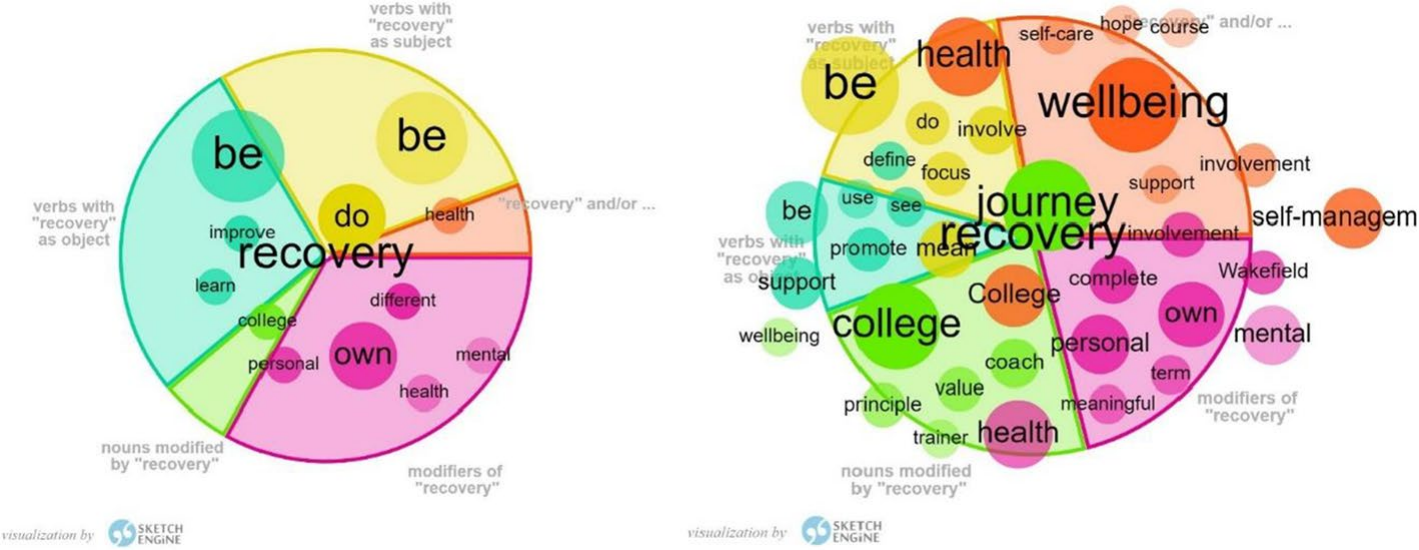
Collocates of “recovery” for RCs in Japan (813 words; left) and England (22,014 words; right). Modifiers are words that describe or add meaning to another word (e.g. recovery in “recovery college”). Colours provided in the figures are automatically generated by Sketch Engine for visual ease

The theme “recovery” was more emphasised in RCs in Japan (Table 3). RCs in Japan and in England showed different contexts for recovery (Fig. 1: word sketch for RCs in Japan and England full datasets). RCs in England characterised recovery as “journey”, “wellbeing”, “self-management” and “personal”. The Japanese dataset was too small to retrieve substantial collocates; however, references to personal, “mental health”, “improve” or being “different” were found. Concordances for recovery were further examined (excluding those for “recovery college” and RC names). Table 6 includes 10 random concordances. RCs in Japan and in England shared understandings of recovery as going beyond the improvement of symptoms and regaining a meaning in life (E21–22, 24, 28). RCs in Japan emphasised the personal dimension (E22) and the importance of the community (E25). RCs in England also acknowledged those aspects; however, a strong focus was placed on recovery as individual change (e.g. learning, acquisition of control and responsibility; E26–27, 30).

**Table 6:** Concordances for recovery

Country	Examples—recovery	
Japan	21	Until now, “recovery” has been considered within a narrow scope and has tended to focus on improving the prognosis of illness…
	22	Recovery here does not simply refer to improvement in mental symptoms or functioning, but rather to living life as the person is
	23	…growing into a person who is content, hopeful, and designing his or her own life at that moment, also known as personal recovery
	24	Recovery is a concept for rebuilding a meaningful and satisfying life despite “difficult life events…
	25	The one thing we all had in common was that recovery does not happen alone, but in a relationship with people you trust
England (full dataset)	26	Recovery is a deeply personal, unique process of changing one’s attitudes, values, feelings, goals, skills and roles
	27	Recovery is a non-linear process of rebuilding after a crisis, taking responsibility for personal wellbeing and learning…
	28	Recovery is a way of living a satisfying, hopeful, and contributing life even with the limitations caused by illness. Recovery involves the development of new meaning and purpose in one's life as one grows beyond the catastrophic effects…
	29	Recovery is holistic and embraces the whole person, including their mind, body, spirit and community
	30	Self-management and self-determination are the foundations of recovery as people define their own goals and plan their own journey towards these goals

### Education

“Education” was emphasised in RCs in England (Table 3), characterised by references to courses, training and the approach adopted. RCs in Japan did not share any of the English RCs’ high-frequency terms related to education (Table 2). Table 7 features five random concordances for “course” (RCs in England), and the only two concordances retrieved for course in RCs in Japan, complemented with concordances for “knowledge”. RCs in England focused on the skills and topics learned (E36–38) and emphasised co-design with people with lived experiences (E39–40). In RCs in Japan, references to “course” focused on co-design (E31-32), and references to knowledge focused on communal learning (E33-35).

**Table 7:** Concordances for “course” and “knowledge”

Country	Examples—course/knowledge	
Japan	31	In addition, the course will be planned, managed, and facilitated together (co-production) by mental health professionals and [people with lived experience]
	32	We also offer courses that draw on the wisdom of our own experiences of mental health difficulties and the expertise of mental health…
	33	Participants learn from each other’s experiences, rather than from a teacher who offers specific knowledge
	34	[people with lived experience] and those who have learned about mental health, called professionals, bring their expertise, knowledge, and wisdom to create a place where they can learn from each other
	35	This is based on the idea that richer possibilities are created when people bring together a variety of experiences and knowledge
England (full dataset)	36	Their educational courses about mental health recovery and self-management are meant to complement existing mental health services
	37	60-min online course about how to recognise loneliness and learn strategies that may help, plus how to increase personal connections…
	38	The Developing Skills and Interests courses aim to develop your skills, interests and confidence, whether it is learning new craft skills, joining in a reading…
	39	Courses and workshops are co-produced with patients, where possible, and run across four areas
	40	Courses are co-designed and co-facilitated by people who have experienced their own mental health challenges, working…

### Community

“Community” was emphasised in RCs in Japan (Table 3). RCs in Japan highlighted community as necessary for personal learning, and identified it as a core aspect of education (Tables 4 and 7). RCs in Japan were characterised as a community space (Table 5), and community was regarded as a central aspect of recovery (Table 6). Table 8 displays the four occurrences of community in RCs in Japan and five random concordances (of 68 in total) for community in RCs in England. RCs in Japan described living in the community as part of recovery (E43–44), whereas descriptions of communities in RCs in England tended to be more specific, e.g. RC partners (E46–48), or those communities that receive support (E45, 47).

**Table 8:** Concordances for “community”

Country	Examples—community	
Japan	41	RC is a learning community where anyone can participate as a student, regardless of position, title or disability to learn about mental health
	42	Recovery College is a place where people with difficulties in living, family members, supporters and community members gather to learn together about living life on their own terms
	43	…those who support them can learn from each other in order to face themselves with sincere joy and live a rich life in the community
	44	…where people with illnesses and their supporters can learn from each other the knowledge they need to live well in the community while coping with various difficulties in their lives
England (full dataset)	45	We support the local community with a particular focus on rehabilitation for individuals who have physical or mental ill health…
	46	We work alongside a range of community partners to bring you the very best opportunities in your local area
	47	This is an exciting and challenging time for practitioners working with vulnerable communities and the Recovery College allows people the time to reflect on hot topics and challenge their thinking…
	48	The core team is made up of a handful of paid staff and volunteers, but the wider team consists of our students, community organisations and other health and social care staff
	49	… supports people to find opportunities to live well within their community

### Answering RQs

To answer RQ1, in both countries, the promotional texts highlighted mental illness experience, reflecting the textual register. In Japan, recovery, wellbeing and community were highlighted, whereas personal learning and education were highlighted in England.

To answer RQ2, the different foci of the promotional texts between the two countries reflect their cultural characteristics. The emphases on recovery as relational and long-term were in line with collectivism and long-term orientation of Japanese culture, relative to English culture. The emphases on personal learning and skill development were in line with individualism and short-term orientation of English culture, relative to Japanese culture.

## Discussion

RCs represent a new mental health recovery approach implemented in 28 countries across diverse cultures. This Japan-England study compared how RCs are introduced to the public. Both countries emphasise lived experiences of mental illness in their promotional texts. Acknowledging lived experience as valid source of knowledge shifts traditional epistemic power imbalances in mental health practices, and it empowers service users. In Japan, RCs accentuated the relational and long-term aspects of recovery, resonating with the cultural characteristics of collectivism and long-term orientation. Conversely, in England, the focus centres on personal learning and skill acquisition, underscoring the relevance to individualism and short-term orientation.

The different emphases evident in promotional texts for RC in Japan and England offer insight into the strategic considerations of RC managers in each country, reflecting cultural values and ideologies. In Japan, emphasis is placed on collectivism and long-term orientation. This strategic emphasis suggests that RC managers in Japan believe that highlighting the collectivistic and long-term aspects of RC will resonate with the public and attract attention. Consequently, it is plausible that individuals attending RCs in Japan may anticipate experiencing and benefiting from these collectivistic and long-term elements. However, these aspects are currently viewed unfavourably within the current RC operational model, assessed as not following the model (Hayes et al., 2023).

The current RC operational model presents challenges for many Asian countries that value collectivism and long-term orientation. For example, in the current RC operational model, there is an expectation for each student to identify and articulate their individual needs. This expectation is underscored in component 3, which evaluates whether an RC actively enquires about the student individual needs (Supplementary Material 2). However, this approach can pose significant difficulties within collectivistic cultures, where individuals are often educated to be attuned to the needs of others, and expressing one’s own needs in a group setting may be considered immature or rude (Schouten et al., 2020). Moreover, RC staff actively enquiring about individual needs may cause stress to students, as it could be experienced as pressuring them to prioritise their own needs over those of the group, leading to potential discomfort or fear of being perceived as different (Kotera et al., 2022). Component 3, as currently formulated, may be unfeasible in Japan and many other Asian countries. In Japanese RCs, for instance, the identification of student individual needs typically occurs in smaller group settings, one-on-one interactions or through online forms. In situations where individual needs are not explicitly expressed, RC staff often resort to introducing past examples or establishing ground rules and activities by reading the atmosphere of the room (“kuki” (空気]) (Meyer, 2022; Uneno et al., 2022). It is imperative that the operational model of RCs incorporates a Collectivistic approach to ensure cultural adaptability in diverse settings.

Likewise, it is essential for the RC operational model to recognise the importance of long-term orientation. Component 7, titled “Commitment to Recovery”, evaluates the presence of positive energy within the RC environment. Assessing this component positively can be difficult for individuals who are long-term-oriented. Long-term orientation views the presence of difficulties as a realistic aspect of life. People oriented to long-term orientation are inclined to believe that encountering challenges is essential for their long-term wellbeing compared to those oriented to short-term orientation (Uchida & Kitayama, 2009). Moreover, people oriented to long-term orientation tend to interpret present difficulties as indicators of future happiness more so than people oriented to short-term orientation (Ji et al., 2001). Furthermore, the term “energy” in component 7 can be interpreted as “qi (気)” in Eastern philosophy, symbolising the importance of holding both yin and yang (positive and negative), inherent in long-term orientation (Feuchtwang, 2016). To ensure fair assessment between long-term versus short-term orientation, adjustments to component 7 are warranted. Such modifications should accommodate the nuanced perspectives and values inherent in long-term orientation, thus fostering equitable evaluation within the RC operational model.

Our analysis did not identify any meaningful differences between Japan and England regarding the other two cultural characteristics associated with the RC operational model: uncertainty acceptance and indulgence. One reason for this may be that these two cultural characteristics are difficult to present in texts, which are a fixed medium of communication. Uncertain acceptance and indulgence are relatively dynamic cultural characteristics (Güss et al., 2017). For example, an uncertain situation is needed to express uncertain acceptance such as decision-making process (Güss et al., 2012). Similarly, indulgence can be observed when someone satisfies (or does not satisfy) their needs such as in healthy behaviour choices (Oh, 2020). To evaluate how these cultural characteristics may manifest in RC operation, future research should engage with more dynamic aspects of RCs such as observing students’ behaviours in RC courses.

The strengths of this study were (a) the first-ever RC research using linguistic analyses; (b) the largest dataset in RC linguistic research (74 RCs, 22,827 words); (c) a Japan-England comparison that can highlight the cultural differences as both countries belong to the Global North, yet Japan is not a WEIRD country (i.e. their economic levels are similar, but cultures are different) and (d) the interpretations of textual analysis according to cultural characteristics, namely, individualism and short-term orientation, were empirically informed, building upon our previous RC studies. This study had several limitations which need to be acknowledged. First, there were several RCs in England that were not included as they did not participate in our RC research programme. Second, we did not investigate how the financial aspects and implementation phases of RCs might impact promotional material. Almost all RCs in England are free to participate, whereas almost all RCs in Japan charge a small fee (e.g. 500 JPY). Moreover, many RCs in England are supported by the National Health Service, whereas many RCs in Japan are supported by non-government organisations. The implementation phases of RCs between Japan and England are also different. Future research should evaluate whether these financial and implementation differences might impact the promotional texts. Third, promotional texts that were not digitised were not collected. Fourth, we compared a non-WEIRD country and a WEIRD country but did not compare a Global-South country and a Global-North country. Fifth, the linguistic differences were not considered. English language is more agentic than Japanese language: English places more emphasis on the causal agent (e.g. a person who caused an event) (Hidaka, 2010; Kotera et al., 2023). However, we believe the impact of the linguistic differences on our findings was not substantial, if any, because the RC promotional texts were not describing accidental events (Fausey et al., 2010). Sixth, we did not focus on different cultural groups in each country (e.g. ethnic minority groups), regional cultural differences (e.g. metropolitan vs rural; seaside vs mountain; the nomenclature of territorial units for statistics in Europe) (Kaasa et al., 2014; Tung, 2008) and different types of cultural characteristics (e.g. horizontal versus vertical individualism (Singelis et al., 1995)). Future research needs to evaluate Global-South countries and different groups, regions and cultural types.

## Conclusion

Using corpus-based discourse analysis, we found cross-cultural differences between Japan and England in RC promotional texts. These differences were in line with cross-cultural biases existed in the RC operational model. Our findings underscore the need for cross-cultural adaptation of the RC operational model. Given the global implementation of RCs, such adaptation could help optimise their effectiveness for individuals in Japan, as well as countries with similar cultural characteristics and multicultural communities.

## Supplementary Information

Below is the link to the electronic supplementary material.

Supplementary file1 (DOCX 80 KB)

## Data Availability

Data are available in the public domain. All sources/URLs are provided in the supplementary materials.
