# Supplementary material for: Cross-cultural Comparison of Recovery College Implementation Between Japan and England: Corpus-based Discourse Analysis
Source: Int J Ment Health Addict. 2024 Jul 5;23(6):4362–86. doi: 10.1007/s11469-024-01356-3 (PMC12698797; doi:10.1007/s11469-024-01356-3)
Supplement: Supplementary file 1 — Supplementary file1 (DOCX 80 KB) [file 11469_2024_1356_MOESM1_ESM.docx]

**Supplementary materials for “Cross-cultural comparison of Recovery College implementation between Japan and England: Corpus-based discourse analysis”**

**Supplementary Material 1**

| Included sources in Bester KL, McGlade A, Darragh E. Is co-production working well in recovery colleges? Emergent themes from a systematic narrative review. *The Journal of Mental Health Training, Education and Practice* 2022; **17**(1): 48-60. | | |
| --- | --- | --- |
|  | **Reference** | **Country** |
| 1 | Cameron J, Hart A, Brooker S, Neale P, Reardon M. Collaboration in the design and delivery of a mental health Recovery College course: experiences of students and tutors. *J Ment Health* 2018; **27**(4): 374-81. | UK |
| 2 | Crowther A, Taylor A, Toney R, et al. The impact of Recovery Colleges on mental health staff, services and society. *Epidemiology and Psychiatric Sciences* 2019; **28**(5): 481-8. | UK |
| 3 | Dalgarno M, Oates J. The meaning of co-production for clinicians: An exploratory case study of Practitioner Trainers in one Recovery College. *Journal of Psychiatric and Mental Health Nursing* 2018; **25**(5-6): 349-57. | UK |
| 4 | McGregor J, Repper J, Brown H. “The college is so different from anything I have done”. A study of the characteristics of Nottingham Recovery College. *The Journal of Mental Health Training, Education and Practice* 2014; **9**(1): 3-15. | UK |
| 5 | Meddings S, Guglietti S, Lambe H, Byrne D. Student perspectives: recovery college experience. *Mental Health and Social Inclusion* 2014; **18**(3): 142-50. | UK |
| 6 | Meddings S, Byrne D, Barnicoat S, Campbell E, Locks L. Co-delivered and co-produced: creating a recovery college in partnership. *The Journal of Mental Health Training, Education and Practice* 2014; **9**(1): 16-25 | UK |
| 7 | Perkins AM, Ridler JH, Hammond L, Davies S, Hackmann C. Impacts of attending recovery colleges on NHS staff. *Mental Health and Social Inclusion* 2017; **21**(1): 18-24. | UK |
| 8 | Sommer J, Gill K, Stein-Parbury J. Walking side-by-side: Recovery Colleges revolutionising mental health care. *Mental Health and Social Inclusion* 2018; **22**(1): 18-26. | Australia |
| 9 | Windsor L, Roberts G, Dieppe P. Recovery Colleges – safe, stimulating and empowering. *Mental Health and Social Inclusion* 2017; **21**(5): 280-8. | UK |
| 10 | Zabel E, Donegan G, Lawrence K, French P. Exploring the impact of the recovery academy: a qualitative study of Recovery College experiences. *The Journal of Mental Health Training, Education and Practice* 2016; **11**(3): 162-71. | UK |

Included sources in Lin E, Harris H, Black G, et al. Evaluating recovery colleges: a co-created scoping review. *Journal of Mental Health* 2022: 1-22.

|  | **Reference** | **Country** |
| --- | --- | --- |
| 1 | Bourne P, Meddings S, Whittington A. An evaluation of service use outcomes in a Recovery College. *Journal of Mental Health* 2018; **27**(4): 359-66. | UK |
| 2 | Burhouse A, Rowland M, Marie Niman H, et al. Coaching for recovery: a quality improvement project in mental healthcare. *BMJ Quality Improvement Reports* 2015; **4**(1): u206576.w2641. | UK |
| 3 | Cameron J, Hart A, Brooker S, Neale P, Reardon M. Collaboration in the design and delivery of a mental health Recovery College course: experiences of students and tutors. *J Ment Health* 2018; **27**(4): 374-81. | UK |
| 4 | Critchley A, Dokter D, Odell-Miller H, Power N, Sandford S. Starting from Scratch: Co-production with dramatherapy in a Recovery College. *Dramatherapy* 2019; **40**(2): 63-80. | UK |
| 5 | Cronin P, Stein-Parbury J, Sommer J, Gill KH. What about value for money? A cost benefit analysis of the South Eastern Sydney Recovery and Wellbeing College. *Journal of Mental Health* 2021: 1-8. | Australia |
| 6 | Dunn EC, Sally Rogers E, Hutchinson DS, et al. Results of an Innovative University-based Recovery Education Program for Adults with Psychiatric Disabilities. *Administration and Policy in Mental Health and Mental Health Services Research* 2008; **35**(5): 357-69. | United States |
| 7 | Durbin A, Nisenbaum R, Wang R, Hwang SW, Kozloff N, Stergiopoulos V. Recovery Education for Adults Transitioning From Homelessness: A Longitudinal Outcome Evaluation. *Frontiers in Psychiatry* 2021; **12**. | Canada |
| 8 | Ebrahim S, Glascott A, Mayer H, Gair E. Recovery Colleges; how effective are they? *The Journal of Mental Health Training, Education and Practice* 2018; **13**(4): 209-18. | UK |
| 9 | Frayn E, Duke J, Smith H, Wayne P, Roberts G. A voyage of discovery: setting up a recovery college in a secure setting. *Mental Health and Social Inclusion* 2016; **20**(1): 29-35. | UK |
| 10 | Hall T, Jordan HL, Reifels L, et al. A Process and Intermediate Outcomes Evaluation of an Australian Recovery College. *Journal of Recovery in Mental Health* 2018; **1**(3): 7-20. | Australia |
| 11 | Harper L, McKeown M. Why make the effort? Exploring recovery college engagement. *Mental Health and Social Inclusion* 2018; **22**(1): 27-33. | UK |
| 12 | Hopkins L, Foster A, Nikitin L. The process of establishing Discovery College in Melbourne. *Mental Health and Social Inclusion* 2018; **22**(4): 187-94. | Australia |
| 13 | Hopkins L, Pedwell G, Lee S. Educational outcomes of Discovery College participation for young people. *Mental Health and Social Inclusion* 2018; **22**(4): 195-202. | Australia |
| 14 | Hopkins L, Foster A, Belmore S, Anderson S, Wiseman D. Recovery colleges in mental health-care services: an Australian feasibility and acceptability study. *Mental Health and Social Inclusion* 2022; **26**(1): 12-22. | Australia |
| 15 | Kay K, Edgley G. Evaluation of a new recovery college: delivering health outcomes and cost efficiencies via an educational approach. *Mental Health and Social Inclusion* 2019; **23**(1): 36-46. | UK |
| 16 | Khan BM, Reid N, Brown R, Kozloff N, Stergiopoulos V. Engaging Adults Experiencing Homelessness in Recovery Education: A Qualitative Analysis of Individual and Program Level Enabling Factors. *Frontiers in Psychiatry* 2020; **11**. | Canada |
| *17 | King T, Meddings S. Survey identifying commonality across international Recovery Colleges. *Mental Health and Social Inclusion* 2019; **23**(3): 121-8. | International |
| 18 | Lucchi F, Chiaf E, Placentino A, Scarsato G. Programma FOR: A Recovery College in Italy. *Journal of Recovery in Mental Health* 2018; **1**(3): 29-37. | Italy |
| 19 | Meddings S, Guglietti S, Lambe H, Byrne D. Student perspectives: recovery college experience. *Mental Health and Social Inclusion* 2014; **18**(3): 142-50. | UK |
| 20 | Meddings S, Campbell E, Guglietti S, et al. From service user to student: The benefits of recovery college. *Clinical Psychology Forum* 2015; (268): 32–7. | UK |
| 21 | Meddings S, Walsh L, Patmore L, McKenzie KLE, Holmes S. To what extent does Sussex Recovery College reflect its community? An equalities and diversity audit. *Mental Health and Social Inclusion* 2019; **23**(3): 136-44. | UK |
| 22 | Muir-Cochrane E, Lawn S, Coveney J, Zabeen S, Kortman B, Oster C. Recovery college as a transition space in the journey towards recovery: An Australian qualitative study. *Nursing & Health Sciences* 2019; **21**(4): 523-30. | Australia |
| 23 | Newman-Taylor K, Stone N, Valentine P, Hooks Z, Sault K. The Recovery College: A unique service approach and qualitative evaluation. *Psychiatr Rehabil J* 2016; **39**(2): 187-90. | UK |
| 24 | Nurser K, Hunt D, Bartlett T. Do recovery college courses help to improve recovery outcomes and reduce self-stigma for individuals who attend? *Clinical Psychology Forum* 2017; **300**: 32–7. | UK |
| 25 | Peer JE, Gardner M, Autrey S, Calmes C, Goldberg RW. Feasibility of implementing a recovery education center in a Veterans Affairs medical center. *Psychiatr Rehabil J* 2018; **41**(2): 135-40. | United States |
| 26 | Reid N, Castel S, Veldhuizen S, Roberts A, Stergiopoulos V. Effect of a Psychiatric Emergency Department Expansion on Acute Mental Health and Addiction Service Use Trends in a Large Urban Center. *Psychiatric Services* 2019; **70**(11): 1053-6. | Canada |
| 27 | Sommer J, Gill K, Stein-Parbury J. Walking side-by-side: Recovery Colleges revolutionising mental health care. *Mental Health and Social Inclusion* 2018; **22**(1): 18-26. | Australia |
| 28 | Sommer J, Gill KH, Stein-Parbury J, Cronin P, Katsifis V. The role of recovery colleges in supporting personal goal achievement. *Psychiatric Rehabilitation Journal* 2019; **42**(4): 394. | Australia |
| 29 | Stevens J, Butterfield C, Whittington A, Holttum S. Evaluation of Arts based Courses within a UK Recovery College for People with Mental Health Challenges. *International Journal of Environmental Research and Public Health*, 2018; **15**(6): 1170. | UK |
| 30 | Sutton R, Lawrence K, Zabel E, French P. Recovery College influences upon service users: a Recovery Academy exploration of employment and service use. *Journal of Mental Health Training, Education & Practice* 2019; **14**(3): 141-8. | UK |
| 31 | Thompson H, Simonds L, Barr S, Meddings S. Recovery colleges: long-term impact and mechanisms of change. *Mental Health and Social Inclusion* 2021; **25**(3): 232-42. | UK |
| 32 | Wilson C, King M, Russell J. A mixed-methods evaluation of a Recovery College in South East Essex for people with mental health difficulties. *Health & Social Care in the Community* 2019; **27**(5): 1353-62. | Canada |
| 33 | Windsor L, Roberts G, Dieppe P. Recovery Colleges – safe, stimulating and empowering. *Mental Health and Social Inclusion* 2017; **21**(5): 280-8. | UK |
| 34 | Zabel E, Donegan G, Lawrence K, French P. Exploring the impact of the recovery academy: a qualitative study of Recovery College experiences. *The Journal of Mental Health Training, Education and Practice* 2016; **11**(3): 162-71. | UK |
| 35 | Secker J, Wilson C. Evaluation of the mid Essex recovery college, October–December 2013. Chelmsford: Anglia Ruskin University, 2014. | UK |
| 36 | Arbour S, Stevens A, Gasparini J. Recovery College: Influencing recovery-related outcomes. Culture and System Transformation [PowerPoint] 2019. | Canada |
| 37 | Barton S, Williams R. Evaluation of “the exchange” (Barnsley Recovery College). https://rfact.org.au/wp-content/uploads/ 2015/05/Evaluation-of-The-Exchange-Barnsley-Recovery-College- SWYFT.pdf 2015. | UK |
| 38 | Canadian Mental Health Association. Recovery College: *White paper*. Canadian Mental Health Association 2020. | Canada |
| 39 | Kaminskiy E. Moore S. *South Essex Recovery College evaluation project report* (pp. 1–53). https://arro.anglia.ac.uk/id/eprint/ 600456 2015. | UK |
| 40 | Kenny B. Kavanagh E. McSherry H. Brady G. Kelly J. MacGabhann L. Griffin M. Farrelly M. Kelly N. Ross P. Barron R. Watters R. Keating S. *Informing and transforming communities, shaping the way forward for mental health recovery*. Dublin North. North East Recovery College 2020. | Ireland |
| 41 | Mayo Recovery College. *My words, my way*. Mayo Recovery College n.d. | Ireland |
| 42 | Taylor D. Boland A. Wallace N. *Mid West ARIES project: A report on the development, progress and outcomes of a pilot project to provide a recovery education service in the Mid West*. University of Limerick 2017. | Ireland |
| 43 | Wels J. *Recovery College Greenwich – Evaluation report*. https://www.bridgesupport.org/wp-content/uploads/2019/01/Enc-4.1- Greenwich-University-Evaluation-Report-August-2016-Final.pdf 2016. | UK |

*Same study as No. 6 in Theriault’s review in 2020 (full reference below), which categorised the location of this study being in England.

Thériault J, Lord M-M, Briand C, Piat M, Meddings S. Recovery Colleges After a Decade of Research: A Literature Review. *Psychiatric Services* 2020; **71**(9): 928-40.

Included sources in Thériault J, Lord M-M, Briand C, Piat M, Meddings S. Recovery Colleges After a Decade of Research: A Literature Review. *Psychiatric Services* 2020; **71**(9): 928-40.

|  | Authors | **Country** |
| --- | --- | --- |
| 1 | Anfossi A. *The current state of Recovery Colleges in the UK*. Nottingham, UK, Implementing Recovery Through Organisational Change 2017 | England |
| 2 | Bourne P, Meddings S, Whittington A. An evaluation of service use outcomes in a Recovery College. *Journal of Mental Health* 2018; **27**(4): 359-66. | England |
| 3 | Chung TE, Eckerle Curwood S, Thang H, Gruszecki S, Beder M, Stergiopoulos V. Introducing a Recovery Education Centre for Adults Experiencing Mental Health Challenges and Housing Instability in a Large Urban Setting. *International Journal of Mental Health and Addiction* 2016; **14**(5): 850-5. | Canada |
| 4 | Dunn EC, Sally Rogers E, Hutchinson DS, et al. Results of an Innovative University-based Recovery Education Program for Adults with Psychiatric Disabilities. *Administration and Policy in Mental Health and Mental Health Services Research* 2008; **35**(5): 357-69. | England |
| 5 | Kay K, Edgley G. Evaluation of a new recovery college: delivering health outcomes and cost efficiencies via an educational approach. *Mental Health and Social Inclusion* 2019; **23**(1): 36-46. | England |
| *6 | King T, Meddings S. Survey identifying commonality across international Recovery Colleges. *Mental Health and Social Inclusion* 2019; **23**(3): 121-8. | England |
| 7 | Meddings S, Campbell E, Guglietti S, et al. From service user to student: The benefits of recovery college. *Clinical Psychology Forum* 2015; (268): 32–7. | England |
| 8 | Meddings S, Walsh L, Patmore L, McKenzie KLE, Holmes S. To what extent does Sussex Recovery College reflect its community? An equalities and diversity audit. *Mental Health and Social Inclusion* 2019; **23**(3): 136-44. | England |
| 9 | Nurser K, Hunt D, Bartlett T. Do recovery college courses help to improve recovery outcomes and reduce self-stigma for individuals who attend? *Clinical Psychology Forum* 2017; **300**: 32–7. | England |
| 10 | Sommer J, Gill KH, Stein-Parbury J, Cronin P, Katsifis V. The role of recovery colleges in supporting personal goal achievement. *Psychiatric Rehabilitation Journal* 2019; **42**(4): 394. | Australia |
| 11 | Burhouse A, Rowland M, Marie Niman H, et al. Coaching for recovery: a quality improvement project in mental healthcare. *BMJ Quality Improvement Reports* 2015; **4**(1): u206576.w2641. | England |
| 12 | Ebrahim S, Glascott A, Mayer H, Gair E. Recovery Colleges; how effective are they? *The Journal of Mental Health Training, Education and Practice* 2018; **13**(4): 209-18. | England |
| 13 | Hall T, Jordan HL, Reifels L, et al. A Process and Intermediate Outcomes Evaluation of an Australian Recovery College. *Journal of Recovery in Mental Health* 2018; **1**(3): 7-20. | Australia |
| 14 | Hopkins L, Pedwell G, Lee S. Educational outcomes of Discovery College participation for young people. *Mental Health and Social Inclusion* 2018; **22**(4): 195-202. | Australia |
| 15 | Lucchi F, Chiaf E, Placentino A, Scarsato G. Programma FOR: A Recovery College in Italy. *Journal of Recovery in Mental Health* 2018; **1**(3): 29-37. | Italy |
| 16 | Meddings S, Guglietti S, Lambe H, Byrne D. Student perspectives: recovery college experience. *Mental Health and Social Inclusion* 2014; **18**(3): 142-50. | England |
| 17 | Perkins AM, Ridler JH, Hammond L, Davies S, Hackmann C. Impacts of attending recovery colleges on NHS staff. *Mental Health and Social Inclusion* 2017; **21**(1): 18-24. | England |
| 18 | Stevens J, Butterfield C, Whittington A, Holttum S. Evaluation of Arts based Courses within a UK Recovery College for People with Mental Health Challenges. *International Journal of Environmental Research and Public Health*, 2018; **15**(6): 1170. | England |
| 19 | Cameron J, Hart A, Brooker S, Neale P, Reardon M. Collaboration in the design and delivery of a mental health Recovery College course: experiences of students and tutors. *J Ment Health* 2018; **27**(4): 374-81. | England |
| 20 | Crowther A, Taylor A, Toney R, et al. The impact of Recovery Colleges on mental health staff, services and society. *Epidemiology and Psychiatric Sciences* 2019; **28**(5): 481-8. | England |
| 21 | Dalgarno M, Oates J. The meaning of co-production for clinicians: An exploratory case study of Practitioner Trainers in one Recovery College. *Journal of Psychiatric and Mental Health Nursing* 2018; **25**(5-6): 349-57. | England |
| 22 | Frayn E, Duke J, Smith H, Wayne P, Roberts G. A voyage of discovery: setting up a recovery college in a secure setting. *Mental Health and Social Inclusion* 2016; **20**(1): 29-35. | England |
| 23 | Gill KH. Recovery colleges, co-production in action: The value of the lived experience in “learning and growth for mental health”. *Health Issues* 2014; (113): 10-4. | Australia |
| 24 | Harper L, McKeown M. Why make the effort? Exploring recovery college engagement. *Mental Health and Social Inclusion* 2018; **22**(1): 27-33. | England |
| 25 | Martina T. Poetry for recovery: Peer trainer reflections at Sussex Recovery College. *Clinical psychology forum* 2015; **268**: 28-31. | England |
| 26 | Newman-Taylor K, Stone N, Valentine P, Hooks Z, Sault K. The Recovery College: A unique service approach and qualitative evaluation. *Psychiatr Rehabil J* 2016; **39**(2): 187-90. | England |
| 27 | Nurser K, Rushworth I, Shakespeare T, Williams D. Personal storytelling in mental health recovery. *Mental Health Review Journal* 2018; **23**(1): 25-36. | England |
| 28 | Sommer J, Gill K, Stein-Parbury J. Walking side-by-side: Recovery Colleges revolutionising mental health care. *Mental Health and Social Inclusion* 2018; **22**(1): 18-26. | Australia |
| 29 | Toney R, Elton D, Munday E, et al. Mechanisms of Action and Outcomes for Students in Recovery Colleges. *Psychiatric Services* 2018; **69**(12): 1222-9. | England |
| 30 | Windsor L, Roberts G, Dieppe P. Recovery Colleges – safe, stimulating and empowering. *Mental Health and Social Inclusion* 2017; **21**(5): 280-8. | England |
| 31 | Zabel E, Donegan G, Lawrence K, French P. Exploring the impact of the recovery academy: a qualitative study of Recovery College experiences. *The Journal of Mental Health Training, Education and Practice* 2016; **11**(3): 162-71. | England |

*Same study as No. 17 in Lin’s review in 2022 (full reference below), which categorised this study as an international study.

Lin E, Harris H, Black G, et al. Evaluating recovery colleges: a co-created scoping review. *Journal of Mental Health* 2022: 1-22.

Included sources in

Crowther A, Taylor A, Toney R, et al. The impact of Recovery Colleges on mental health staff, services and society. *Epidemiology and Psychiatric Sciences* 2019; **28**(5): 481-8., and

Toney R, Elton D, Munday E, et al. Mechanisms of Action and Outcomes for Students in Recovery Colleges. *Psychiatric Services* 2018; **69**(12): 1222-9. (Same 44 sources)

|  | **Authors** | **Country** |
| --- | --- | --- |
| 1 | Frayn E, Duke J, Smith H, Wayne P, Roberts G. A voyage of discovery: setting up a recovery college in a secure setting. *Mental Health and Social Inclusion* 2016; **20**(1): 29-35. | UK |
| 2 | P erkins R, Repper J. When is a “recovery college” not a “recovery college”? *Mental Health and Social Inclusion* 2017; **21**(2): 65-72. | UK |
| 3 | Meddings S, Campbell E, Guglietti S, et al. From service user to student: The benefits of recovery college. *Clinical Psychology Forum* 2015; (268): 32–7. | UK |
| 4 | Taggart H, Kempton J. The Route to Employment: The Role ofMental Health Recovery Colleges. London: CentreForum; 2015. | UK |
| 5 | North Essex Research Network. Evaluation of the Mid Essex Recovery College October–December 2013. Essex: North Essex Research Network; 2014. | UK |
| 6 | The Dorset Wellbeing and Recovery Partnership (WaRP). WaRP Magazine. 2016. | UK |
| 7 | McGregor J, Repper J, Brown H. “The college is so different from anything I have done”. A study of the characteristics of Nottingham Recovery College. *The Journal of Mental Health Training, Education and Practice* 2014; **9**(1): 3-15. | UK |
| 8 | Oh H. The pedagogy of recovery colleges: clarifying theory. *Mental Health Review Journal* 2013; **18**(4). | UK |
| 9 | Skipper L, Page K. Our recovery journey: two stories of change within Norfolk and Suffolk NHS Foundation Trust. *Mental Health and Social Inclusion* 2015; **19**(1): 38-44. | UK |
| 10 | Watson E. What Makes a Recovery College? A Systematic LiteratureReview of Recovery Education in Mental Health [Dissertation]. Nottingham: University of Nottingham; 2013. | UK |
| 11 | Kelly J, Gallagher S, McMahon J. Developing a recovery college: a preliminary exercise in establishing regional readiness and community needs. *Journal of Mental Health* 2017; **26**(2): 150-5. | UK |
| 12 | Dunn EC, Sally Rogers E, Hutchinson DS, et al. Results of an Innovative University-based Recovery Education Program for Adults with Psychiatric Disabilities. *Administration and Policy in Mental Health and Mental Health Services Research* 2008; **35**(5): 357-69. | UK |
| 13 | Hall T, Brophy L, Jordan H. A report on the early outcomes of the Mind Recovery College. Melbourne: Centre for Mental Health, Melbourne School of Population and Global Health, 2016. | Australia |
| 14 | Hall T, Brophy L, Jordan H, et al. Co-Producing The Journey To Recovery: The Mind Recovery College, Australia. TheMHS Conference 2016; 2016; Auckland, Australia: TheMHS Learning Network Inc.; 2016. | Australia |
| 15 | McGregor J, Brophy L, Hardy D, et al. Proceedings of June 2015 Meeting. Recovery Colleges International Community of Practice (RCICoP); 2015; 2015. | UK |
| 16 | Mind. Australasian Recovery College Community of Practice Inaugural Meeting. Victoria: Mind; 2016. | Australia |
| 17 | Newman-Taylor K, Stone N, Valentine P, Hooks Z, Sault K. The Recovery College: A unique service approach and qualitative evaluation. *Psychiatr Rehabil J* 2016; **39**(2): 187-90. | UK |
| 18 | Shepherd G, McGregor J. Recovery Colleges – Evolution or Revolution?. Ghent, November 9; 2016. | UK |
| 19 | Sussex Recovery College. Performance and Evaluation Report (Summer Term 2016); Brighton: Sussex Partnership NHS Foundation Trust; 2016 | UK |
| 20 | Thornhill H, Dutta A. Are recovery colleges socially acceptable? *BJPsych International* 2016; **13**(1): 6-7. | UK |
| 21 | Zabel E, Donegan G, Lawrence K, French P. Exploring the impact of the recovery academy: a qualitative study of Recovery College experiences. *The Journal of Mental Health Training, Education and Practice* 2016; **11**(3): 162-71. | UK |
| 22 | Burhouse A, Rowland M, Marie Niman H, et al. Coaching for recovery: a quality improvement project in mental healthcare. *BMJ Quality Improvement Reports* 2015; **4**(1): u206576.w2641. | UK |
| 23 | Central and North West London NHS Foundation Trust (2015) CNWL Recovery & Wellbeing College Annual Report April 2014 - July 2015. | UK |
| 24 | Kaminskiy E, Moore S. South essex recovery college evaluation. Chelmsford, UK.: Anglia Ruskin University, 2015. | UK |
| 25 | King T. An exploratory study of co-production in recovery colleges in the UK. Sussex: University of Brighton.; 2015. | UK |
| 26 | Meddings S, Campbell E, Guglietti S, et al. From service user to student: The benefits of recovery college. *Clinical Psychology Forum* 2015; (268): 32–7. | UK |
| 27 | Gill KH. Recovery colleges, co-production in action: The value of the lived experience in “learning and growth for mental health”. *Health Issues* 2014; (113): 10-4. | Australia |
| 28 | McCaig M, McNay L, Marland G, Bradstreet S, Campbell J. Establishing a recovery college in a Scottish University. *Mental Health and Social Inclusion* 2014; **18**(2): 92-7. | UK |
| 29 | McMahon J, Wallace N, Kelly J, Egan E. Recovery education college: a needs analysis. Dublin: Health Service Executive, 2014. | Ireland |
| 30 | Meddings S, Byrne D, Barnicoat S, Campbell E, Locks L. Co-delivered and co-produced: creating a recovery college in partnership. *The Journal of Mental Health Training, Education and Practice* 2014; **9**(1): 16-25. | UK |
| 31 | Meddings S, Guglietti S, Lambe H, Byrne D. Student perspectives: recovery college experience. *Mental Health and Social Inclusion* 2014; **18**(3): 142-50. | UK |
| 32 | Rennison J, Skinner S, Bailey A. CNWL Recovery College Annual Report April 2013 - March 2014. London: Central and North West London NHS Foundation Trust., 2014. | UK |
| 33 | Alois Zucchelli F, Skinner S. Central and North West London NHS Foundation Trust's (CNWL) Recovery College: the story so far …. *Mental Health and Social Inclusion* 2013; **17**(4): 183-9. | UK |
| 34 | Mind. Establishment of the Mind Recovery College. Heidelberg: Mind Australia, 2012. | Australia |
| 35 | Perkins R, Repper J, Rinaldi M, Brown H. Recovery Colleges. London: Implementing Recovery Through Organisational Change, 2012. | UK |
| 36 | Rinaldi M, Morland M, Wybourn S. Annual Report 2011 – 2012 South West London Recovery College, London, South West London and St George’s Mental Health NHS Trust. London: National Health Service, 2012. | UK |
| 37 | Rinaldi M, Suleman M. Care co-ordinators’ attitudes to self-management and their experience of the use of the South West London Recovery College. London: South West London and St George's Mental Health NHS Trust.; 2012 | UK |
| 38 | Rinaldi M, Wybourn S. The Recovery College Pilot in Merton and Sutton: longer term individual and service level outcomes. London: South West London and St. Georges Mental Health NHS Trust.; 2011. | UK |
| 39 | Bourne P, Meddings S, Cooper R, Locks L, Whittington A. An evaluation of service use outcomes in Sussex Recovery College. . Sussex: Sussex NHS Trust; 2016. | UK |
| 40 | Bristow E. An annual report of Lincoln Recovery College. Lincoln: Lincolnshire Partnership NHS Foundation Trust, 2015. | UK |
| 41 | Martina T. Poetry for recovery: Peer trainer reflections at Sussex Recovery College. *Clinical psychology forum* 2015; **268**: 28-31. | UK |
| 42 | Solent Recovery College. Solent Recovery College, Our first year – Outcomes. . Portsmouth: Solent Recovery College; 2014. | UK |
| 43 | Barton, South West Yorkshire Partnership NHS Foundation Trust Recovery College [PowerPoint] n.d. | UK |
| 44 | Sault, Garner, Gatherer, Southern Health Recovery College [PowerPoint] n.d. | UK |

Included sources in Toney R, Knight J, Hamill K, et al. Development and Evaluation of a Recovery College Fidelity Measure. *The Canadian Journal of Psychiatry* 2018; **64**(6): 405-14

| **No** | **Authors** | **Country** |
| --- | --- | --- |
| 1 | McGregor J, Repper J, Brown H. “The college is so different from anything I have done”. A study of the characteristics of Nottingham Recovery College. *The Journal of Mental Health Training, Education and Practice* 2014; **9**(1): 3-15. | UK |
| 2 | Perkins R, Repper J, Rinaldi M, Brown H. Briefing: Recovery Colleges2012. https://imroc.org/wp-content/uploads/2022/04/1.Recovery-Colleges.pdf (accessed 23 January 2023). | UK |
| 3 | Mind. Australasian Recovery College Community of Practice Inaugural Meeting. Victoria: Mind; 2016. | Australia |
| 4 | Meddings S, Campbell E, Guglietti S, et al. From service user to student: The benefits of recovery college. *Clinical Psychology Forum* 2015; (268): 32–7. | UK |
| 5 | Shepherd G, McGregor J, Meddings S, Roeg W. Recovery colleges and co-production. *Wellbeing, recovery and mental health* 2017: 181-93. | UK |
| 6 | Perkins AM, Ridler JH, Hammond L, Davies S, Hackmann C. Impacts of attending recovery colleges on NHS staff. *Mental Health and Social Inclusion* 2017; **21**(1): 18-24. | UK |
| 7 | Shepherd G, Boardman J, Rinaldi M, Roberts G. Supporting recovery in mental health services: Quality and outcomes. *London: Implementing Recovery Through Organisational Change* 2014. | UK |
| 8 | Leamy M, Bird V, Boutillier CL, Williams J, Slade M. Conceptual framework for personal recovery in mental health: systematic review and narrative synthesis. *British Journal of Psychiatry* 2011; **199**(6): 445-52. | UK |
| 9 | McGregor J, Brophy L, Hardy D, et al. Proceedings of June 2015 Meeting. Recovery Colleges International Community of Practice (RCICoP); 2015; 2015. | UK |
| 10 | Rinaldi M, Morland M, Wybourn S. Annual Report 2011 – 2012 South West London Recovery College, London, South West London and St George’s Mental Health NHS Trust. London: National Health Service, 2012. | UK |
| 11 | Watson E. What Makes a Recovery College? A Systematic LiteratureReview of Recovery Education in Mental Health [Dissertation]. Nottingham: University of Nottingham; 2013. | UK |
| 12 | Meddings S, Guglietti S, Lambe H, Byrne D. Student perspectives: recovery college experience. *Mental Health and Social Inclusion* 2014; **18**(3): 142-50. | UK |
| 13 | King T. An exploratory study of co-production in recovery colleges in the UK. Sussex: University of Brighton.; 2015. | UK |

**Supplementary Material 2**

Recovery College Operational Components (Toney et al., 2018)

| **No** | **Component** | **Definition** |
| --- | --- | --- |
| 1 | Valuing equality | The contributions and assets of students, trainers (peers, clinicians, external) and other staff are equally valued. No one is judged or treated differently because of their background or mental health difficulties. |
| 2 | Learning | Recovery Colleges follow an adult education approach whereby students and trainers collaborate and learn from each other by sharing experiences, knowledge and skills. Students have responsibility for their learning and learn through interactive and reflective exercises. Students gain self-awareness, understanding of their difficulties and practical, relevant self-management skills. Students choose courses which best suit their interests and aspirations. |
| 3 | Tailored to the student | Recovery Colleges don’t offer a one-size-fits-all experience. Students’ individual needs are actively enquired about and accommodated during courses (e.g. personalised handouts, translated text, materials adapted for learning difficulties). Their needs outside the course are also accommodated (e.g. buddy service, transport help, individual learning plans). |
| 4 | Co-production of the Recovery College | People with lived experience (Peer Trainers and students) are brought together with professionals and subject experts to design and deliver all aspects of the Recovery College. This includes collaborative decision-making about the prospectus, courses, college policies, staff recruitment, advertising, etc., as well as the co-design and co-delivery of all courses by a Peer Trainer and other subject-expert. |
| 5 | Social connectedness | Both the culture and the physical environment of the college provide students with opportunities to develop connections with others. The learning space is relaxed, e.g. non-clinical chair layout, access to drinks facilities, shared spaces for socialising. Trainers recognise and cater for students' social needs, e.g. organising exercises and breaks for chatting, sharing experiences and developing friendships. |
| 6 | Community focus | Recovery Colleges engage with community organisations (e.g. mental health charities, artistic/sporting groups) and Further Education colleges to coproduce relevant courses. The college provides students with information, handouts and events which support students' pathways into valued activities, roles, relationships and support in the community. |
| 7 | Commitment to recovery | Recovery College workers talk with conviction and enthusiasm about the service and are dedicated to students' recovery. There is a positive energy in the college and its activities, based on shared values about the recovery principles on which the college is based. |

**Supplementary Material 3**

A list of methodological terms

| Collocates | Words that co-occur with a focus word (or node) more frequently than would be expected by chance. |
| --- | --- |
| Concordance tool | A corpus linguistics tool that allows the user to search for individual words or phrases and retrieves all their occurrences in a given corpus together with the context of use. Results are retrieved in the form of concordances, strings of texts with the search terms in the middle, and can be counted, filtered and sorted in different manners. |
| Keyness | The numerical measure that establishes the uniqueness of a particular word or phrase within the dataset studied (focus corpus) in relation to a reference dataset (reference corpus) based on statistics. The numerical value that expresses the degree of keyness is the keyness score. |
| Keyword lists | A corpus linguistics analysis tool that automatically retrieves words that are more frequent in the dataset studied (focus corpus) in comparison to another dataset of reference (reference corpus) that is usually (but not necessarily) larger. Keywords provide insights into what is specific or unique of the dataset considered in relation to another. |
| Lemma | The standard form that is the form you see in the dictionary e.g., “learn” instead of “learned” or “learning” etc. |
| Sketch Engine | Corpus linguistics analysis software that helps understand how words and phrases are used in the real-world language by providing automatic extraction of typical or rare words from the dataset under study, patterns of co-occurring words, and detailed information of how words are used in context (Kilgarriff et al., 2014). Sketch Engine is available at: https://www.sketchengine.eu/. |
| Word Sketch | A corpus linguistics analysis tool developed by the Sketch Engine project. Word Sketch identifies words co-occurring with a focus word (i.e., collocates, see above) and provides information about how the focus word and the collocates ‘behave’ in the dataset analysed. This means that the tool provides an overview of grammatical relations and allows users to identify patterns of use (i.e., how a word fits into sentences, what other words it tends to be found alongside, and what meanings and evaluations are associated with it) based on statistics. |
| Wordlist | A corpus linguistics analysis tool that compiles lists of words from a text or collection of texts (corpus) to perform analyses such as frequency analysis. This tool provides insights into the different words used in a text (both lexical and grammatical terms), and their raw and relative (normalised) frequencies. This tool can help us understand recurrent vocabulary used in the texts and is useful for various linguistic and educational purposes. |

**Supplementary Material 4**

Full dataset of Recovery Colleges in England (N=61, sub-set highlighted in grey, n=4)

| **Recovery College Name** | **Words** | **URL** |
| --- | --- | --- |
| [Digital Recovery College](https://www.dualdiagnosis.org.uk/recovery-college-listings/st-mungos-recovery-college-now-digital-recovery-college/) | 288 | <https://www.nottinghamshirehealthcare.nhs.uk/nottingham-recovery-college/> |
| [Severn and Wye Recovery College](https://www.swrecoverycollege.nhs.uk/) | 243 | <https://www.swrecoverycollege.nhs.uk/> |
| [Oxfordshire R](https://oxfordshirerecoverycollege.org.uk/what-are-recovery-colleges/)ecovery College | 231 | <https://oxfordshirerecoverycollege.org.uk/what-are-recovery-colleges/> |
| [Leicestershire Recovery College](https://www.leicspart.nhs.uk/service/leicestershire-recovery-college/) | 89 | <https://www.leicspart.nhs.uk/service/leicestershire-recovery-college/> |
| [Sussex Recovery College](https://www.sussexrecoverycollege.org.uk/) | 1,414 | <https://www.sussexrecoverycollege.org.uk/> |
| [Devon Recovery Learning Community](https://devonrlc.co.uk/) | 975 | <https://devonrlc.co.uk/> |
| [Humber Recovery and Wellbeing College](https://humberrecoverycollege.nhs.uk/adult) | 896 | <https://humberrecoverycollege.nhs.uk/adult> |
| [City and Hackney Recovery College](https://www.dualdiagnosis.org.uk/recovery-college-listings/city-and-hackney-recovery-college-previously-city-and-hackney-mind-recovery-college/) | 760 | <https://www.dualdiagnosis.org.uk/recovery-college-listings/city-and-hackney-recovery-college-previously-city-and-hackney-mind-recovery-college/> |
| [Greater Manchester Mental Health NHS Trust Recovery Academy](https://www.gmmh.nhs.uk/recovery/) | 743 | <https://www.gmmh.nhs.uk/recovery/> |
| [The Exchange Recovery College](https://www.barnsleyrecoverycollege.nhs.uk/) | 600 | <https://www.barnsleyrecoverycollege.nhs.uk/> |
| [South West London Recovery College](https://www.swlstg.nhs.uk/south-west-london-recovery-college) | 593 | <https://www.swlstg.nhs.uk/south-west-london-recovery-college> |
| [Wakefield Recovery & Wellbeing College](http://www.wakefieldrecoverycollege.nhs.uk/what-is-a-recovery-college/) | 589 | <http://www.wakefieldrecoverycollege.nhs.uk/what-is-a-recovery-college/> |
| [Pennine Care NHS Foundation Trust Health and Wellbeing College](https://hwcollege.penninecare.nhs.uk/) | 568 | <https://hwcollege.penninecare.nhs.uk/> |
| [Involvement, Recovery & Wellness Centre](https://www.cwp.nhs.uk/our-services/east-cheshire/recovery-and-wellness-centre-cheshire-east) | 563 | <https://www.cwp.nhs.uk/our-services/east-cheshire/recovery-and-wellness-centre-cheshire-east> |
| [Recovery in Mind](https://recoveryinmind.org/our-research-project/) | 545 | <https://recoveryinmind.org/our-research-project/> |
| [Waltham Forest Recovery College](https://www.dualdiagnosis.org.uk/recovery-college-listings/waltham-forest-recovery-college/) | 541 | <https://www.dualdiagnosis.org.uk/recovery-college-listings/waltham-forest-recovery-college/> |
| [Bristol Wellbeing College](http://www.second-step.co.uk/wellbeing-colleges/bristol-wellbeing-college/) | 509 | <http://www.second-step.co.uk/wellbeing-colleges/bristol-wellbeing-college/> |
| [Buckinghamshire Recovery College](https://www.oxfordhealth.nhs.uk/bucksrecoverycollege/) | 505 | <https://www.oxfordhealth.nhs.uk/bucksrecoverycollege/> |
| [CNWL Recovery & Wellbeing College](https://www.cnwl.nhs.uk/services/recovery-and-wellbeing-college/what-do-we-mean-recovery) | 495 | <https://www.cnwl.nhs.uk/services/recovery-and-wellbeing-college/what-do-we-mean-recovery> |
| [Northumberland Recovery College](https://northumberlandrecoverycollege.co.uk/about-us/) | 460 | <https://northumberlandrecoverycollege.co.uk/about-us/> |
| [NT LIFE Recovery College](https://voda.org.uk/ntlife/) | 443 | <https://voda.org.uk/ntlife/> |
| [The Recovery College - Southern Health Foundation Trust](https://www.southernhealth.nhs.uk/our-services/a-z-list-of-services/recovery) | 439 | <https://www.southernhealth.nhs.uk/our-services/a-z-list-of-services/recovery> |
| [ARCH Recovery College](https://www.tewv.nhs.uk/get-involved/training/arch-recovery-college/) | 423 | <https://www.tewv.nhs.uk/get-involved/training/arch-recovery-college/> |
| [Recovery College Greenwich](https://www.therecoveryplace.co.uk/) | 420 | <https://www.therecoveryplace.co.uk/> |
| [Discovery Centre](https://www.dpt.nhs.uk/our-services/secure-care/discovery-centre) | 408 | <https://www.dpt.nhs.uk/our-services/secure-care/discovery-centre> |
| [RCE Wellbeing Hub](https://www.cpft.nhs.uk/rce-wellbeing-hub) | 381 | <https://www.cpft.nhs.uk/rce-wellbeing-hub> |
| [West London Recovery College](https://www.westlondon.nhs.uk/our-services/adult/mental-health-services/recovery-college) | 377 | <https://www.westlondon.nhs.uk/our-services/adult/mental-health-services/recovery-college> |
| [The Life Rooms](https://www.liferooms.org/) | 369 | <https://www.liferooms.org/> |
| [Calderdale and Kirklees Recovery and Wellbeing College](https://www.calderdalekirkleesrc.nhs.uk/) | 368 | <https://www.calderdalekirkleesrc.nhs.uk/> |
| [New Leaf Recovery and Wellbeing College](https://www.newleafcollege.co.uk/) | 368 | <https://www.newleafcollege.co.uk/> |
| [South London and Maudsley NHS Foundation Trust - SLaM Recovery College](https://www.slamrecoverycollege.co.uk/) | 350 | <https://www.slamrecoverycollege.co.uk/> |
| [Inclusion Recovery College Thurrock](https://recoverycollege.inclusionthurrock.org/what-is-recovery-college/) | 348 | <https://recoverycollege.inclusionthurrock.org/what-is-recovery-college/> |
| [Northern Recovery College](https://ads-uk.org/recovery-college/) | 314 | <https://ads-uk.org/recovery-college/> |
| [Lancashire Recovery College](https://www.communityroots.lscft.nhs.uk/) | 301 | <https://www.communityroots.lscft.nhs.uk/> |
| Surrey and North East Hampshire Recovery College | 295 | <https://humberrecoverycollege.nhs.uk/adult> |
| [East Lancashire Recovery College](https://thegreenhouses.org/how-we-help/) | 290 | <https://thegreenhouses.org/how-we-help/> |
| [Recovery College NHFT](https://www.nhft.nhs.uk/recovery-college/) | 287 | <https://www.nhft.nhs.uk/recovery-college/> |
| [ACL Essex Mental Health Recovery Programme](https://aclessex.com/mental-health/#:~:text=Fee%3A%20Free-,In%20the%20ACL%20Mental%20Health%20and%20Wellbeing%20Programme%2C%20this%206,and%20strategies%20to%20achieve%20an) | 286 | <https://aclessex.com/mental-health/> |
| [Cornwall Recovery College](https://www.pentreath.co.uk/recovery-college-cornwall/about-recovery-college-cornwall/) | 263 | <https://www.pentreath.co.uk/recovery-college-cornwall/about-recovery-college-cornwall/> |
| [Help for Heroes Recovery College](https://www.helpforheroes.org.uk/get-help/recovery-college/) | 244 | <https://www.helpforheroes.org.uk/get-help/recovery-college/> |
| [Clarendon Recovery College](https://www.haringey.gov.uk/social-care-and-health/mental-health/clarendon-recovery-college) | 241 | <https://www.haringey.gov.uk/social-care-and-health/mental-health/clarendon-recovery-college> |
| [Recovery & Every Day Skills Recovery College](https://www.stah.org/patients/reds#:~:text=At%20REDS%20Recovery%20College%20we,better%20hopes%20for%20the%20future.) | 240 | <https://www.stah.org/patients/reds> |
| [Dorset Recovery Education Centre](https://www.dorsethealthcare.nhs.uk/patients-and-visitors/our-services-hospitals/REC) | 230 | <https://www.dorsethealthcare.nhs.uk/patients-and-visitors/our-services-hospitals/REC> |
| [Wokingham Recovery College](https://www.wokingham.gov.uk/health/health-services-and-advice/wokingham-recovery-college-mental-health-support) | 229 | <https://www.wokingham.gov.uk/health/health-services-and-advice/wokingham-recovery-college-mental-health-support> |
| [Tower Hamlets Recovery College](https://www.elft.nhs.uk/tower-hamlets-recovery-college/about-recovery-college) | 218 | <https://www.elft.nhs.uk/tower-hamlets-recovery-college/about-recovery-college> |
| [Bedfordshire and Luton Recovery College](https://www.elft.nhs.uk/services/bedfordshire-and-luton-recovery-college) | 212 | <https://www.elft.nhs.uk/services/bedfordshire-and-luton-recovery-college> |
| [Recovery College for All](https://www.bsmhft.nhs.uk/service-users-and-carers/service-user-information/recovery/recovery-college-for-all/) | 190 | <https://www.bsmhft.nhs.uk/service-users-and-carers/service-user-information/recovery/recovery-college-for-all/> |
| [Solent Recovery College](https://www.solentmind.org.uk/support-for-you/our-services/solent-recovery-college/) | 170 | <https://www.solentmind.org.uk/support-for-you/our-services/solent-recovery-college/> |
| [Brighton & Hove Recovery College](https://www.southdown.org/services/recovery-college-brighton-hove/) | 165 | <https://www.southdown.org/services/recovery-college-brighton-hove/> |
| [Nottingham Recovery College](https://www.nottinghamshirehealthcare.nhs.uk/nottingham-recovery-college/) | 164 | <https://www.nottinghamshirehealthcare.nhs.uk/nottingham-recovery-college/> |
| [Lincolnshire Recovery College](https://www.lpft.nhs.uk/our-services/adults/recovery-college) | 160 | <https://www.lpft.nhs.uk/our-services/adults/recovery-college> |
| [ReCoCo](https://www.recoverycoco.com/about) | 155 | <https://www.recoverycoco.com/about> |
| [Compass Recovery College](https://www.compassrecoverycollege.uk/) | 153 | <https://www.compassrecoverycollege.uk/> |
| [Recovery and Wellbeing Academy](https://www.recoveryandwellbeing.co.uk/) | 147 | <https://recoveryinmind.org/our-research-project/> |
| [Leeds Recovery College](http://www.mindwell-leeds.org.uk/services-directory/leeds-recovery-college/) | 137 | <http://www.mindwell-leeds.org.uk/services-directory/leeds-recovery-college/> |
| [Wellbeing and Recovery College](https://recovery.mpft.nhs.uk/) | 126 | <https://recovery.mpft.nhs.uk/> |
| [REACH Recovery College](https://www.trustlinks.org/projects/reach-recovery-college/) | 126 | <https://www.trustlinks.org/projects/reach-recovery-college/> |
| [Mind in Bexley - Recovery College](https://mindinbexley.org.uk/recovery/) | 115 | <https://mindinbexley.org.uk/recovery/> |
| [Camden and Islington Recovery College](https://www.candi.nhs.uk/our-services/education-and-employment/recovery-college) | 100 | <https://www.candi.nhs.uk/our-services/education-and-employment/recovery-college> |
| [Kent & Medway Recovery & Wellbeing College](https://www.kmpt.nhs.uk/about-us/recovery-and-wellbeing-college/) | 97 | <https://www.kmpt.nhs.uk/about-us/recovery-and-wellbeing-college/> |
| [The north Cumbria Recovery College](https://wcmhp.org.uk/index.php/ova_sev/north-cumbria-recovery-college/) | 58 | <https://wcmhp.org.uk/index.php/ova_sev/north-cumbria-recovery-college/> |
| TOTAL | 22014 |  |

**Supplementary Material 5**

Full dataset of Recovery Colleges in Japan (N=13)

| **Recovery College Name** | **Words** | **URL** |
| --- | --- | --- |
| RC Mitaka | 113 | <https://sudachikai.eco.to/pia/about_us.html> |
| RC Ohta | 113 | <https://sites.google.com/edu.teu.ac.jp/recoveryota> |
| RC Kochi | 94 | <https://linktr.ee/rc_kochi> |
| RC Kobe | 88 | <https://rcchauchaukobe.jimdofree.com/> |
| RC Tanto | 88 | <https://www.recoverycollegetanto.com/> |
| RC Okayama | 75 | <https://rcokayama.jp/about/recoverycollege> |
| RC Fukuoka | 62 | <https://www.rcfukuoka.com/> |
| RC Nagoya | 50 | [https://recoverycollege-nagoya.com/](https://recoverycollege-nagoya.com/%ef%bd%92%ef%bd%83%e3%81%ab%e3%81%a4%e3%81%84%e3%81%a6/) |
| RC Saga | 45 | <https://peatix.com/event/3590660?fbclid=IwAR3bepl4JVaKxh_TwKqKDiL1neNTO0cjwf6fidPMw7Yu8Sg-i641qZHCiVo> |
| RC Annaka | 37 | <https://www.facebook.com/recoverycollege.annaka/mentions> |
| RC Mimasaka | 21 | <https://www.facebook.com/photo?fbid=2339223402964977&set=pcb.2334556536764997> |
| RC Neyagawa | NA* | <https://rcneyagawa.blog.fc2.com/> |
| TOTAL | 813 |  |

**Supplementary Material 6**

Adapted version of the analytical framework for Critical Discourse Analysis (Fairclough, 2010, 2014; Mullet, 2018)

| **Stage of analysis** | **Description** | **How it is addressed in our work** |
| --- | --- | --- |
| Preparation (i): select the discourse | Select a discourse according to your research interests.  Originally focused on addressing social injustices and inequalities, (critical) discourse analysis is also applied to discourses that promote positive social change (Bartlett, 2017; Martin, 2004) | Selection of Recovery Colleges (RCs) promotional texts, a relatively new mental health support system that promotes individual empowerment and recovery through learning in community. |
| Preparation (ii): data gathering | Select data sources, consider any ethical implications involved in data gathering, and prepare the data for analysis. | Identification of RCs in England and Japan, and retrieval of relevant descriptions from their information websites, notably focusing on those presenting RCs and recovery to the public.  Translation of Japan RCs descriptions into English.  Preparation of two .TXT files (Japan RCs and England RCs datasets) to be used with the software Sketch Engine. |
| Text analysis (micro-level) | According to research interests: Identify the major underlying themes and subthemes.  Examine linguistic choices used to represent social actors or events.  Examine the stance taken by the author/speaker.  Examine whether the text includes references to other texts (intertextuality). | Corpus linguistics-based analysis supported with the software Sketch Engine to retrieve keywords (single and multi-words) and identification of key themes for each dataset.  Examination of concordances (keywords in context) guided by the RQs (i.e., construal of RCs and recovery in England and Japan RCs promotional texts). Focus on both the portrayal of the RCs and the role attribution to service users. |
| Discourse practice  (meso-level) | Examine the contexts of production and reception of the text. Consider the goal of the text, who has produced it, and the putative audience. | Context of production (RCs as mental health intervention) and values of producers (RC managerial staff) and putative audience (RC students) have been studied (Kotera et al., 2024). For the purpose of this study (i.e., construal of RC and recovery), the linguistic analysis has not considered characteristics of the textual register (promotional texts of medical services). |
| Sociocultural practice  (macro-level) | Consider relevant sociocultural or historical factors (i.e., context of production) that have conditioned the text. Consider whether the text reflects any sociocultural values. | Examination of RCs as new mental health support system and its main underpinning philosophies (see Section 1 Introduction).  Interpretation of the main themes emerging from the linguistic analysis based on Hofstede's cultural dimensions theory (Hofstede & Minkov, 2013) and Kotera et al.’s study on the impact of culture on the RC operational model (Kotera et al., 2024). |

**References**

Bartlett, T. (2017). Positive discourse analysis 1. In J. Flowerdew & J. E. Richardson (Eds.), *The Routledge handbook of critical discourse studies* (pp. 133-147). Routledge. <https://doi.org/https://doi.org/10.4324/9781315739342>

Fairclough, N. (2010). *Critical discourse analysis* (2 ed.). Routledge.

Fairclough, N. (2014). *Language and Power* (3 ed.). Routledge.

Hofstede, G., & Minkov, M. (2013). *Values Survey Module 2013 Manual*. Geert Hofstede BV. <https://geerthofstede.com/wp-content/uploads/2016/07/Manual-VSM-2013.pdf>

Kilgarriff, A., Baisa, V., Bušta, J., Jakubíček, M., Kovář, V., Michelfeit, J., Rychlý, P., & Suchomel, V. (2014, 2014/07/01). The Sketch Engine: ten years on. *Lexicography, 1*(1), 7-36. <https://doi.org/10.1007/s40607-014-0009-9>

Kotera, Y., Ronaldson, A., Hayes, D., Hunter-Brown, H., McPhilbin, M., Dunnett, D., Jebara, T., Takhi, S., Masuda, T., Camacho, E., Bakolis, I., Repper, J., Meddings, S., Stergiopoulos, V., Brophy, L., De Ruysscher, C., Okoliyski, M., Kubinová, P., Eplov, L., Toernes, C., Narusson, D., Tinland, A., Puschner, B., Hiltensperger, R., Lucchi, F., Miyamoto, Y., Castelein, S., Borg, M., T, G., Meng, R., Sornchai, C., Tiengtom, K., Farkas, M., Moreland, H., Moore, E., Butler, A., Mpango, R., Tse, S., Kondor, Z., Ryan, M., Zuaboni, G., Elton, D., Grant-Rowles, J., McNaughton, R., Hanlon, C., Harcla, C., Vanderplasschen, W., Arbour, S., Silverstone, D., Bejerholm, U., Ling, C., Ochoa, S., Garcia-Franco, M., Tolonen, J., Yeo, C., Charles, A., Henderson, C., & Slade, M. (2024). How culture impacts recovery intervention: 28-country global study on associations between cultural characteristics and Recovery College fidelity. *Preprint*. <https://doi.org/10.13140/RG.2.2.34787.36648>

Martin, J. R. (2004). Positive discourse analysis: Solidarity and change. *Revista Canaria de Estudios Ingleses, 49*, 179-200.

Mullet, D. R. (2018, 2018/05/01). A General Critical Discourse Analysis Framework for Educational Research. *Journal of Advanced Academics, 29*(2), 116-142. <https://doi.org/10.1177/1932202X18758260>

Toney, R., Knight, J., Hamill, K., Taylor, A., Henderson, C., Crowther, A., Meddings, S., Barbic, S., Jennings, H., Pollock, K., Bates, P., Repper, J., & Slade, M. (2018, 2019/06/01). Development and Evaluation of a Recovery College Fidelity Measure. *The Canadian Journal of Psychiatry, 64*(6), 405-414. <https://doi.org/10.1177/0706743718815893>
